# Immunopathological Profile of Patients with Thymic Epithelial Tumour and Good Syndrome in Advanced Stage

**DOI:** 10.1007/s10875-026-02050-w

**Published:** 2026-07-04

**Authors:** Fabiana Napolitano, Erica Pietroluongo, Michele Francesco Di Tolla, Margaret Ottaviano, Vittoria D’Esposito, Pietro De Placido, Alberto Servetto, Giuseppe Portella, Roberto Bianco, Giovannella Palmieri, Pietro Formisano, Anna Maria Malfitano

**Affiliations:** 1 Department of Translational Medical Sciences, University of Naples Federico II, Via Sergio Pansini 5, Naples, 80131 Italy; 2 Department of Clinical Medicine and Surgery, University of Naples Federico II, Naples, Italy; 3 Department of Medicine, Section of Hematology Oncology, Thoracic Oncology Program, The University of Chicago Medicine, Chicago, IL USA; 4 Department of Melanoma, Cancer Immunotherapy and Development Therapeutics, Istituto Nazionale Tumori IRCCS- Fondazione “G. Pascale”, Naples, Italy; 5 Institute of Endotypes in Oncology, Metabolism and Immunology “G. Salvatore” - National Research Council (IEOMI-CNR), Naples, Italy; 6 Department of Advanced Biomedical Sciences, University of Naples Federico II, Via Pansini 5, Naples, 80131 Italy; 7 Broad Institute of MIT and Harvard, Cambridge, MA USA; 8 Rare Tumors Coordinating Center of Campania Region (CRCTR), Naples, Italy

**Keywords:** Thymic epithelial tumours, Good syndrome, Autoimmune diseases, Immunoglobulins, Immunophenotype, Cytokines

## Abstract

**Purpose:**

Thymic epithelial tumors (TETs) are associated with Good Syndrome (GS), a secondary immunodeficiency characterized by hypogammaglobulinemia, B-cell lymphopenia, and recurrent infections. This study investigated the immunological profile of TET patients to identify immune alterations associated with GS, independently of autoimmune diseases (AD) and disease stage.

**Methods:**

Seventy patients with TETs were stratified according to GS status (GS+/GS−), AD status (AD+/AD−), and disease stage (advanced disease, IVA/B, or no evidence of disease, NED). Serum immunoglobulins, peripheral immune cell subsets, and circulating cytokines, chemokines, growth factors, and metabolic markers were evaluated.

**Results:**

GS+ patients showed reduced IgM and IgG levels compared with GS- patients; however, after stratification, only IgM remained reduced in GS+AD− NED patients. Peripheral B-cell lymphopenia was consistently observed in GS+ patients across both disease stages, independently of AD status. Treg frequencies increased in GS− patients with advanced disease and in GS+AD+ NED patients. Monocytopenia and an inverted CD4+/CD8+ ratio were mainly observed in GS+AD+ patients with advanced disease. Cytokine profiling identified increased levels of IL-1β, IL-10, IL-17, TNFα, IFN-γ, IL-4, IL-5, IL-6, Eotaxin, G-CSF, and IP-10 in GS+ patients. Among these, IP-10 remained consistently elevated regardless of disease stage or AD status.

**Conclusion:**

Peripheral B-cell lymphopenia and elevated IP-10 levels represent the most consistent immunological features associated with GS, independent of disease stage and AD status, whereas serum immunoglobulin levels appear less reliable in advanced disease. These findings support the diagnostic value of immunophenotyping, particularly B-cell quantification and IP-10 measurement, for identifying GS in patients with TETs.

**Supplementary Information:**

The online version contains supplementary material available at https://doi.org/10.1007/s10875-026-02050-w.

## Introduction

Thymomas, thymic carcinomas, and thymic neuroendocrine tumors are rare tumors classified as thymic epithelial tumors (TETs). TETs originate from the thymus, the key organ for the development of the immunological repertoire. B and T lymphocytes originate from a common precursor in the bone marrow (BM). B cells, after completing their maturation in the BM, enter the circulation, while T cell precursors migrate to the thymus for T lymphopoiesis [1]. According to the World Health Organization (WHO), TETs are classified into thymomas, subdivided into type A, AB, and B1-B3, and thymic carcinomas. Staging is defined according to the TNM classification system. Early-stage disease (stage I–II) includes tumors limited to the thymus or minimally invading surrounding mediastinal fat or pericardium, with no lymphnode involvement or distant metastases. Advanced stages (stage III–IV) feature macroscopic invasion of adjacent structures (stage III), pleural or pericardial dissemination (stage IVA), or hematogenous metastases with deep lymphnode involvement (stage IVB). The TNM system was recently updated to its 9th edition, refining early-stage classifications while keeping advanced-stage definitions unchanged [2, 3].

In early-stage TETs, particularly thymomas, complete surgical resection remains the primary treatment modality and the most significant prognostic factor, with 5-year overall survival rates exceeding 85–90%. In selected cases, adjuvant radiotherapy or chemotherapy may be considered as part of a multimodal approach. Conversely, in advanced-stage disease, especially thymic carcinomas, complete resection is often not feasible, and systemic chemotherapy, sometimes in conjunction with radiotherapy, is used predominantly for palliative intent [4, 5]. However, the rarity of these tumors makes the development of alternative therapeutic strategies challenging.

TETs are frequently associated with autoimmune diseases (AD) [6] and immunodeficiency [7, 8]. Myasthenia gravis (MG) is the most common AD detected in thymoma patients; however, aplastic anemia, pure red cell aplasia (PRCA), Hashimoto’s thyroiditis, autoimmune hemolytic anemia, systemic lupus, lichen planus pemphigus, are also frequently reported [9]. In addition, Good syndrome (GS), a parathymic syndrome within the spectrum of AD and immunodeficiency has an incidence of 0.2%–6% in thymoma [10] and is characterized by hypogammaglobulinemia secondary to thymoma, deficiency of serum immunoglobulins (Ig) (IgA, IgM and IgG), B-cell lymphopenia, inversion of the CD4+/CD8 + T cell ratio, and downregulation of Treg lymphocytes [11]. The main cause of death in GS patients is attributed to frequent bacterial, fungal, and opportunistic infections [8]. Ig replacement therapy reduces the mortality rate in GS, and immunosuppressive treatments may be administered to GS patients if their immunological status is accurately monitored [9]. Immunological mechanisms responsible for humoral and cell-mediated alterations suggest aberrant T-cell subtypes and cytokine dysregulation [12]. In a previous study, we observed high lymphocyte blood levels, marked B-cell lymphopenia and downregulation of Treg in TET GS+ patients with associated AD (GS + AD+ TET) compared to TET patients without AD (GS + AD- TET). In GS + AD+ TET patients, we detected also increased serum levels of IL-6, IL-15, IP-10, VEGF, GM-CSF, and MIP-1α, overall, our study highlighted the need to monitor the immunophenotype of TET patients to improve their clinical management [9].

In this study, we investigated differences in Ig levels, immunophenotype, and circulating cytokines, chemokines and growth factors in TET patients with or without GS and in the presence/absence of concomitant AD, the differences were evaluated in disease advanced stage IVA and IVB (IVA/B) and in patients with no evidence of disease (NED).

## Materials and Methods

### Study Design and Participants

Consecutive patients with TETs referred to the Regional Coordinating Center for Rare Tumors (CRCTR) of Campania Region, at University Hospital Federico II (Naples, Italy), were enrolled from 2019 to 2023. Thymus neoplasia was classified according to the World Health Organization (WHO) classification, and tumour stage was determined according to the TNM staging system, 8th edition [13]. Histological subtype classification was based on surgical resection specimens in all patients who underwent thymectomy. In the remaining patients who did not undergo surgical resection (unresectable advanced disease), classification was based on core-needle or CT-guided biopsy material. The designation of “thymoma not otherwise specified (NOS)” was applied when the available tumor material (biopsy specimen) was insufficient to allow definitive subclassification according to WHO criteria, precluding assignment to a specific type (A, AB, B1, B2, or B3) [14].

GS was defined by hypogammaglobulinemia, B-cell lymphopenia, CD4 + T-cell lymphopenia, abnormal CD4+/CD8 + ratio, impaired T-cell mitogenic responses, and recurrent infections caused by encapsulated bacteria, fungi, and viruses [8, 10, 11]. Other AD were defined as clinically documented immune-mediated disorders diagnosed at baseline or at any time during follow-up, based on specialist evaluation and disease-specific diagnostic criteria, guidelines, or validated scoring systems, as applicable to each condition.

Polyautoimmunity was defined as the co-occurrence of two or more distinct AD in the same patient [15]. Blood samples were collected at different time points during the clinical course, including follow-up visits in NED patients and disease assessment visits in patients with advanced-stage disease (IVA/IVB). Patients were treatment-free for at least 3 weeks before blood sampling. The types of treatments are reported in Supplementary Table 1. For the aims of this study, 70 patients with TETs were recruited.

### Serum Levels of Ig

In 52 patients, the serum levels of IgA, IgM and IgG were monitored using a nephelometer (Siemens instruments) with BNII and BN Prospect, using reagents (DADE Behring). Ig were measured in 26 patients with GS (GS+) and in 26 without GS (GS-). A further stratification was performed dividing each group in two subgroups at the date of blood sampling: patients at stage IVA/B and patients with NED. Then, patients were further divided considering the presence or the absence of other AD, resulting in the following subgroups for IVA/B and NED patients: GS + AD+, GS + AD-, GS-AD+, and GS-AD- patients.

### Whole Blood Lysis and Staining

In 70 patients, the immunophenotype was evaluated, measuring B lymphocytes, monocytes, neutrophils, eosinophils, natural killer (NK), CD4 + and CD8 + T cells by 8-color immunophenotyping kit (Miltenyi Biotec) as previously described [9]. Briefly, 100 µl of whole blood from each patient were stained with 10 µl cocktail containing anti-CD3PE, anti-CD4 VioBright 667, anti-CD8APC-Vio770, anti-CD14VioBlue, anti-CD16VioBrigh t515, anti-CD19PE-Vio770, anti-CD45VioGreen, anti-CD56VioBright 515. 7-AAD staining solution was added and after 10 min incubation (in the dark at room temperature), red blood cells were lysed and the resuspended cell pellet was subjected to flow cytometry acquisition (BD LSRFortessa, BD Biosciences, San Jose, CA, USA). The analyses were performed using Flowlogic Software (Milteny Biotech). Similar methods were adopted for Treg detection using a staining cocktail containing anti-CD4FITC, anti-CD25APC, anti-CD45Vio Blue, anti-CD127PE (Treg detection kit: CD4/CD25/CD127, Miltenyi Biotec) as previously described [9]. The immunophenotypic evaluation was performed in 37 GS+ patients and 33 GS- patients. As described above, each group was stratified according to disease stage (IVA/B or NED) and further subdivided based on the presence or absence of AD. The number of patients in each group is indicated in the figures.

### Cytokine Quantification

In 38 patients, serum samples were screened for the concentration of interleukin (IL)-1β, IL-1ra, IL-2, IL-4, IL-5, IL-6, IL-7, IL-8, IL-9, IL-10, IL-12 (p70), IL-13, IL-15, IL-17 A, Eotaxin, basic Fibroblast Growth Factor (bFGF), Granulocyte-Colony Stimulating Factor (G-CSF), Granulocyte and Macrophage-Colony Stimulating Factor (GM-CSF), Interferon-γ (IFN-γ), Interferon-γ inducible Protein-10 (IP-10), Monocyte chemoattractant Protein-1 (MCP-1), Macrophage Inflammatory Protein-1 alpha (MIP-1α), MIP-1β, C-C motif chemokine ligand 5/Regulated on Activation, Normal T-cell Expressed and Secreted (CCL5/RANTES), Tumor Necrosis Factor-α (TNFα), Platelet-Derived Growth Factor-bb (PDGF-bb), and Vascular Endotelial Growth Factor (VEGF), using the Bio-Plex^®^ Multiplex Human Cytokine, Chemokine, and Growth Factor Kit (cat. number M500KCAF0Y, Bio-Rad, Hercules, Ca, USA), as previously described for multiplex cytokine quantification methodology [16]. Concentration values for IL-12 (p70), IL-13, IL-15 and VEGF were not readable for more than 20% of the patients and therefore were excluded from further analyses. Additionally, a second array of metabolic markers, including C-Peptide, Ghrelin, Gastric Inhibitory Peptide (GIP), Glucagon-Like Peptide-1 (GLP-1), Glucagon, Insulin, Leptin, total Plasminogen Activator Inhibitor-1 (PAI-1), Resistin, and Visfatin, was screened using the Bio-Plex Pro^®^ Human Diabetes 10-plex Assay (cat. number 171A7001M, Bio-Rad, Hercules, Ca, USA), as previously described for bead-based biomarker detection procedures [17]. Both magnetic bead-based assays were performed on a Bio-Plex 200 analyzer with the Bio-Rad Bio-Plex Manager software (Bio-Rad, Hercules, CA, United States).

### Ethical Aspects

The study was approved by the Ethical Committee of University of Naples Federico II (approval n.76.21). All enrolled patients signed an informed consent at the study entrance.

### Statistical Analysis

Statistical analysis was performed using both GraphPad^®^ Prism 8.4.2 software (GraphPad Software Inc, La Jolla, Ca, USA) and the R version 4.1.2 programming language (R Foundation for Statistical Computing, Vienna, Austria) through the RStudio^®^ Integrated Development Environment (Posit PBC, Boston, Ma, USA).

Categorical variables were described as the number of occurrences and relative frequency as percentage. Comparisons between two categorical variables were analyzed via Fisher’s exact test. Continuous variables were described as mean ± standard error of mean (SEM), unless differently specified. Shapiro-Wilk normality test was used to evaluate whether the continuous data were normally distributed. For two-group comparisons, Welch’s two-tailed t-test for independent samples (for parametric data) or Mann-Whitney U-test (for non-parametric data) were used. Multiple comparisons among more than two groups were assessed using either ANOVA test with Tukey’s post hoc correction (for parametric data) or Kruskal-Wallis test with Dunn’s post hoc correction (for non-parametric data). All p-values < 0.05 were considered statistically significant.

## Results

### Clinical Characteristics of Patients

Clinical features and treatments of TET patients are reported in Table 1 and in Supplementary Table 1. Among the 70 enrolled patients, 37 (53%) were classified as GS+. GS+ patients had a mean age of 57.3 years, 51% were male, and 70% presented with advanced-stage disease (Masaoka-Koga stage IVA/B). In GS+ patients, respiratory tract infections were the most common infectious complications (78%), followed by urinary tract infections (24%), skin infections (11%), and gastrointestinal infections (5%). Other infectious events, including viral conjunctivitis and keratitis, were observed in 3% of patients. Hospitalization due to infectious complications was required in 24% of cases. Regarding causative pathogens, bacterial infections were the most frequent (86%), followed by viral infections (19%) and opportunistic infections (8%).

**Table 1:** Clinical features of TET patients. The table reports the clinical features of TET patients recruited in this study (age, sex, thymectomy, histological type, stage of disease according to TNM staging, other AD, second primary cancer, types of infections

Patient characteristics	Number of patients (*N* = 70)
Age (years)	
Mean (median)	57.3
Range	31–79
Sex, *n* (%)	
Male	34 (49%)
Female	36 (51%)
Thymectomy, *n* (%)	
Yes	42 (60%)
No	28 (40%)
Histological type, *n* (%)	
Thymoma	48 (69%)
A	6 (9%)
A B	14 (20%)
B1	7 (10%)
B2	7 (10%)
B1-B2	0 (0%)
B2-B3	4 (6%)
B3	11 (16%)
Not otherwise specified	1 (1%)
Thymic carcinoma	19 (27%)
Stage of disease (TNM staging), *n* (%)	
IVA/IVB	43 (61%)
NED	27 (39%)
AD, *n* (%)	
Myasthenia gravis	20 (29%)
PRCA	1 (1%)
Thyroiditis	4 (6%)
Poly-autoimmunity	8 (11%)
Other	5 (7%)
Second primary cancer, *n* (%)	
Kaposi’s sarcoma	2 (3%)
Prostate	2 (3%)
Breast	1 (1%)
Lung	1 (1%)
Other	8 (11%)

### Evaluation of IgA, IgM and IgG

**Fig. 1:**
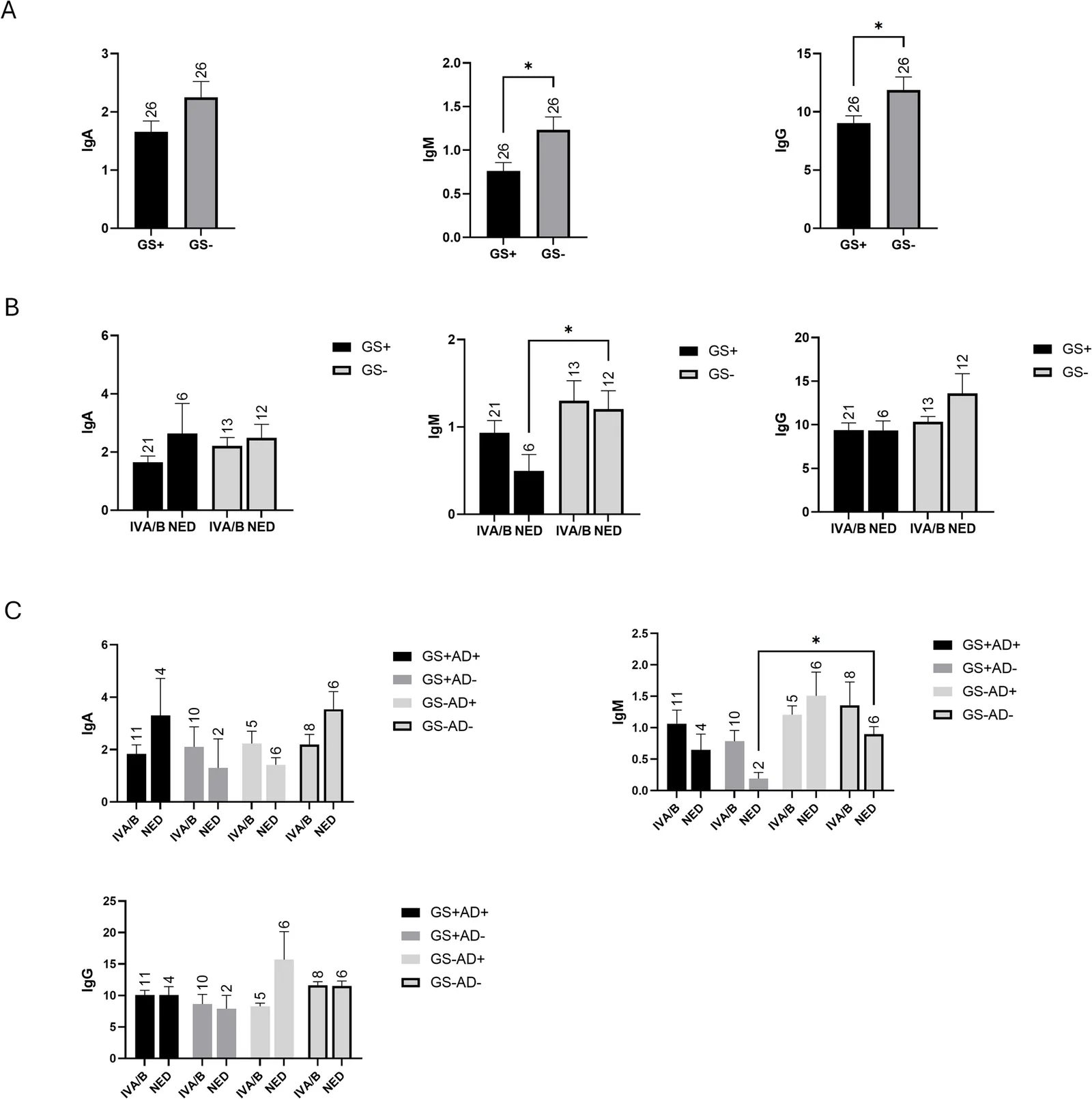
IgA, IgM and IgG levels in TET patients. Ig levels were evaluated in GS + and GS- TET patients (**A**), in IVA/B and NED patients stratified by GS status (**B**), and in IVA/B and NED patients stratified by GS and AD status (**C**). In particular, IgM and IgG levels in GS+ patients were 0.8 ± 0.1 and 9.0 ± 0.6, respectively (**A**). After stratification, IgM levels were significantly lower in GS + NED (0.5 ± 0.2) compared with GS-NED (1.2 ± 0.2) patients (**B**). This reduction was maintained in NED patients when comparing GS + AD- (0.2 ± 0.1) with GS-AD- (0.9 ± 0.1) patients (**C**). Results are illustrated as bar graphs, and the number of patients is indicated above each bar. Bars indicate mean ± SEM. Statistical significance was evaluated by unpaired non-parametric Mann-Whitney U test *p < 0.05

At blood sampling the levels of IgA, IgM and IgG were initially measured in GS + and GS- patients. Overall, GS+ patients showed lower Ig levels compared with GS− patients, with significant differences observed for IgM and IgG (Fig. 1A). After stratification of patients in IVA/B and NED stages, the reduction in IgM levels remained evident only in NED patients, whereas no significant differences were observed in disease progression (Fig. 1B). Additionally, patients were further stratified according to the presence or absence of AD into the following groups: GS + AD+, GS + AD-, GS-AD + and GS-AD-. To assess if GS effect was independent of AD, we compared GS + AD+ vs. GS-AD + and GS + AD- vs. GS-AD-. GS effects were considered independent of AD when differences were observed in both comparisons. As expected, no differences were observed in IgA and IgG levels when comparing GS + AD+ vs. GS-AD + and GS + AD- vs. GS-AD-patients in both IVA/B and NED stages (Fig. 1C). Notably, reduced IgM levels were maintained only in GS + AD- NED patients compared with GS-AD- NED patients (Fig. 1C). However, the subgroup GS + AD- NED included a limited number of patients (2 patients) and therefore should be interpreted cautiously.

### Immune Cell Subset Identification and Quantification

**Fig. 2:**
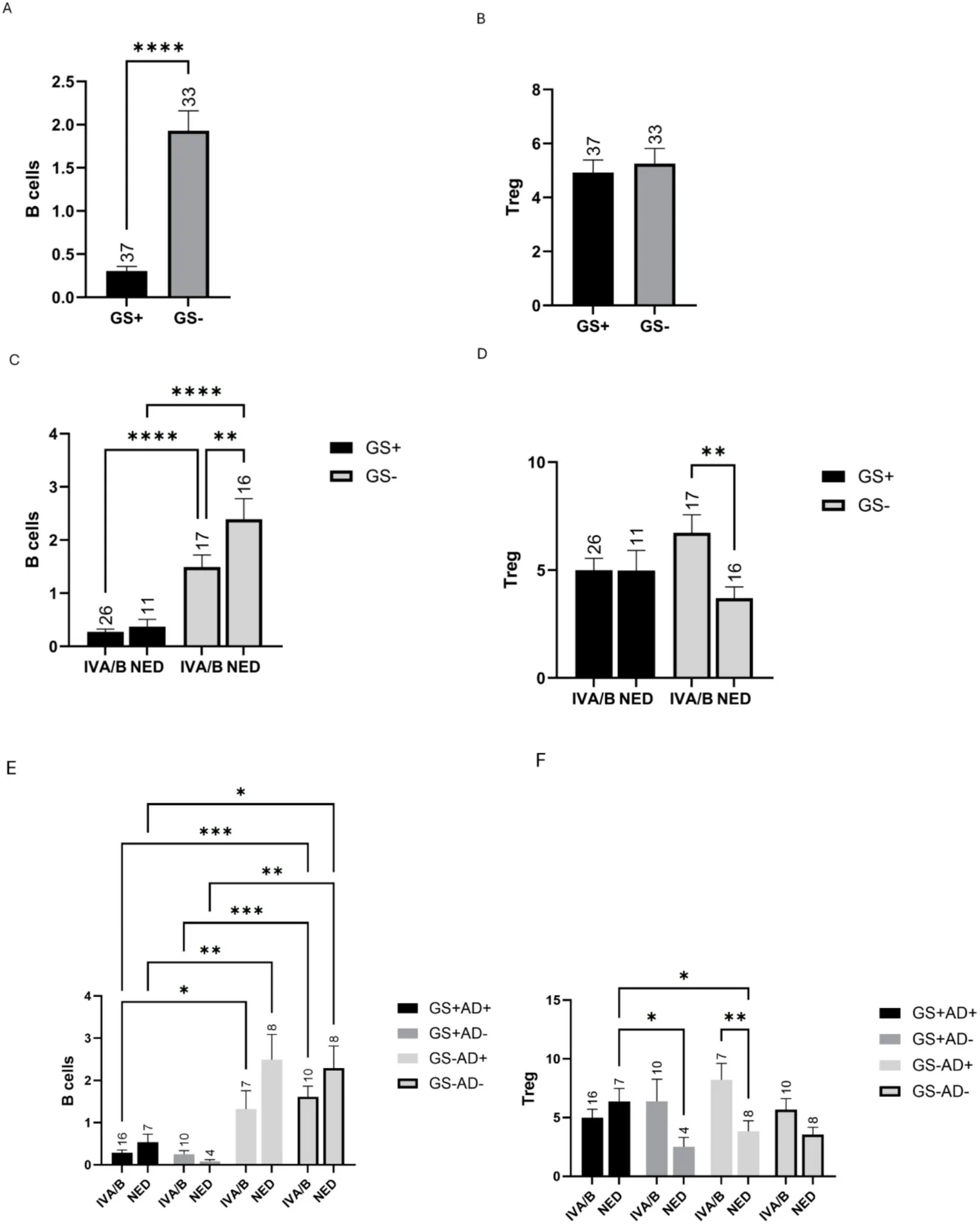
B cells and Treg levels in TET patients. B cells (**A**) and Treg (**B**) were evaluated in GS + and GS- TET patients, in IVA/B and NED patients stratified by GS status (**C**, **D**), and in IVA/B and NED patients stratified by GS and AD status (**E**, **F**). The mean value of B cells in GS+ compared with GS- patients was 0.3 ± 0.1 and 1.9 ± 0.2, respectively (**A**). In GS+ patients, B-cell lymphopenia was confirmed in both IVA/B (0.2 ± 0.1) and NED (0.4 ± 0.1) groups (**C**), whereas in GS- patients higher Treg levels were observed in IVA/B compared with NED (6.7 ± 0.8 vs. 3.7 ± 0.5) (**C**). Results are illustrated as bar graphs, and the number of patients is indicated above each bar. Bars indicate mean ± SEM. Statistical significance was evaluated by Mann-Whitney U test (**A**, ****p < 0.0001); ordinary one-way Anova (**C**, data passed D’Agostino-Pearson normality test, **p < 0.05, ****p < 0.0001); and non-parametric Kruskal-Wallis test (**D**, **p < 0.005; **E**-**F** *p < 0.05, **p < 0.01, ***p < 0.005)

Immune profiling analyses were performed in 70 patients to evaluate B cells, Treg, monocytes, and CD4 + and CD8 + T cells. GS+ patients showed a marked peripheral B-cell lymphopenia compared with GS− patients (Fig. 2A), whereas no overall differences were observed for Treg frequencies (Fig. 2B). Stratification according to disease stage confirmed the persistence of B-cell lymphopenia in GS+ patients across both IVA/B and NED groups (Fig. 2C). No difference was reported in Treg frequencies between GS + and GS-groups (Fig. 2D). However, in advanced stage GS- patients, Treg frequencies increased compared with NED patients (Fig. 2D). Further stratification according to AD status, showed higher B-cell lymphopenia comparing either IVA/B or NED, respectively in GS + AD+ vs. GS-AD+, and GS + AD- vs. GS-AD-, thus supporting an AD-independent effect (Fig. 2E). Treg analysis showed increased levels mainly in GS + AD+ NED patients, whereas advanced-stage GS- patients, particularly those with associated AD, displayed higher Treg frequencies compared with NED patients (Fig. 2F).

**Fig. 3:**
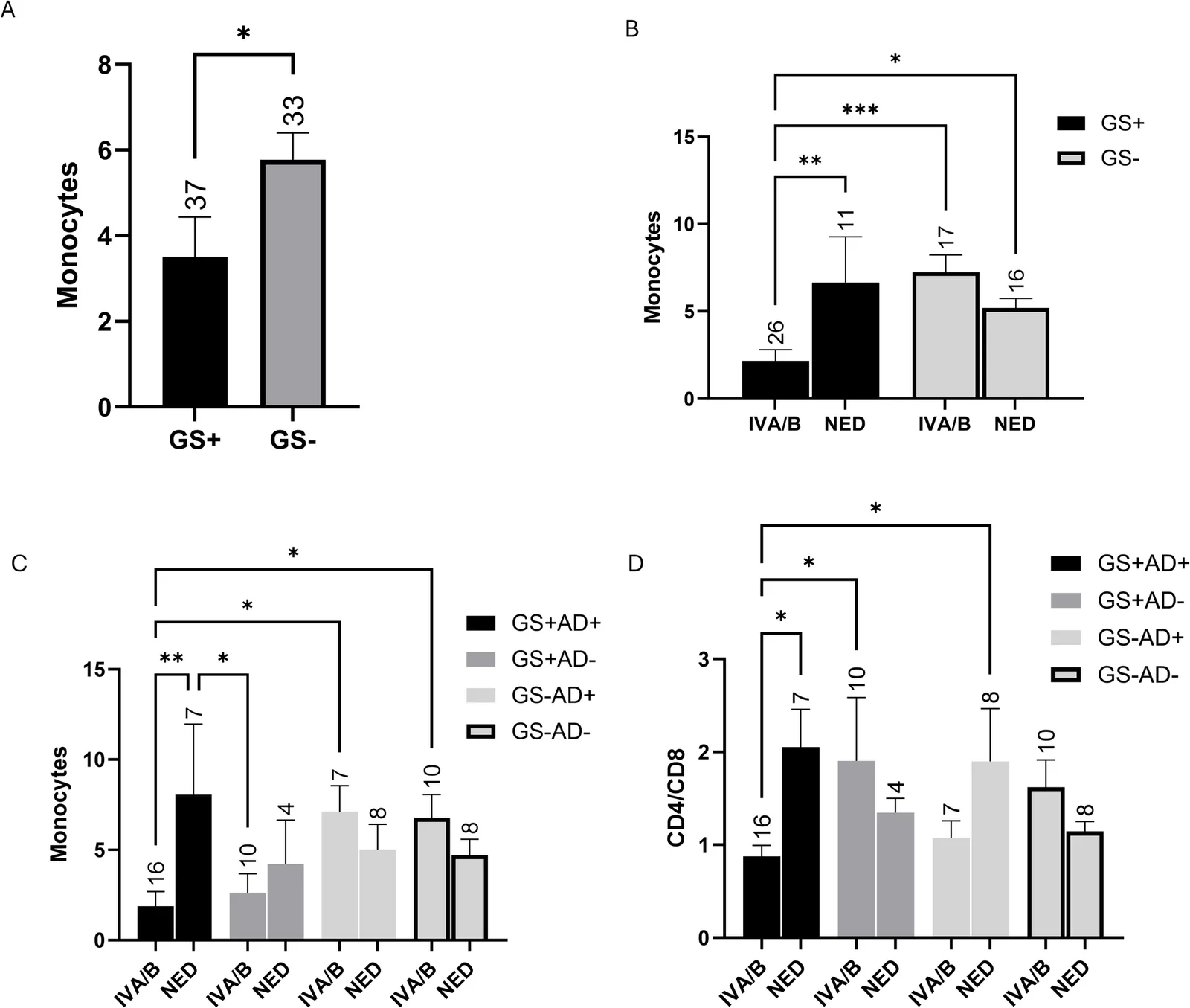
Analyses of monocytes and CD4+/CD8 + ratio in TET patients. Monocytes were evaluated in GS + and GS- TET patients (**A**) in IVA/B and NED patients stratified by GS status (**B**), and in IVA/B and NED patients stratified by both GS and AD status (**C**). CD4+/CD8 + ratio was evaluated (**D**) as described for monocytes (**C**). Reduced levels of monocytes were observed in GS+ (3.5 ± 0.9) compared with GS- (5.8 ± 0.6) patients (A) and remained low in IVA/B (2.2 ± 0.6) patients compared with the other groups (**B**, **C**). Results are reported as bar graphs, and the number of patients is indicated above each bar. Bars indicate mean ± SEM. Statistical significance was evaluated by one-way Anova test, followed by Tukey post-hoc comparison. All p-values < 0.05 were considered significant (*<0.05, ** <0.01, *** <0.001)

Similar analyses were performed for monocytes, neutrophils, eosinophils, NK cells and CD4 + and CD8 + T cells. No significant differences were observed for neutrophils, eosinophils, and NK cells (data not shown). By contrast, monocyte levels were significantly reduced in GS+ patients (Fig. 3A), particularly in advanced-stage disease (Fig. 3B) and in GS + AD+ IVA/B patients (Fig. 3C). Interestingly, monocytopenia in GS + AD+ NED was significantly higher when compared with both IVA/B of GS + AD+ and GS + AD- patients (Fig. 3C). Moreover, the CD4+/CD8 + ratio was inverted mainly in GS + AD+ IVA/B patients (0.9 ± 0.1) compared with GS + AD+ NED, GS-AD + NED, and GS + AD- IVA/B patients (Fig. 3D).

Parallel analyses based on AD status alone did not reveal major differences between AD + and AD- patients, except for B-cell lymphopenia observed in AD+ compared with AD- patients that was maintained in advanced-stage AD+ patients (Supplementary Fig. 1, S1). Similar alterations were detected evaluating the CD4+/CD8 + ratio that was inverted in advanced-stage AD+ patients (Fig.S1), suggesting that major immune alterations were primarily associated with GS rather than AD.

### Cytokine Level Modulation

**Fig. 4:**
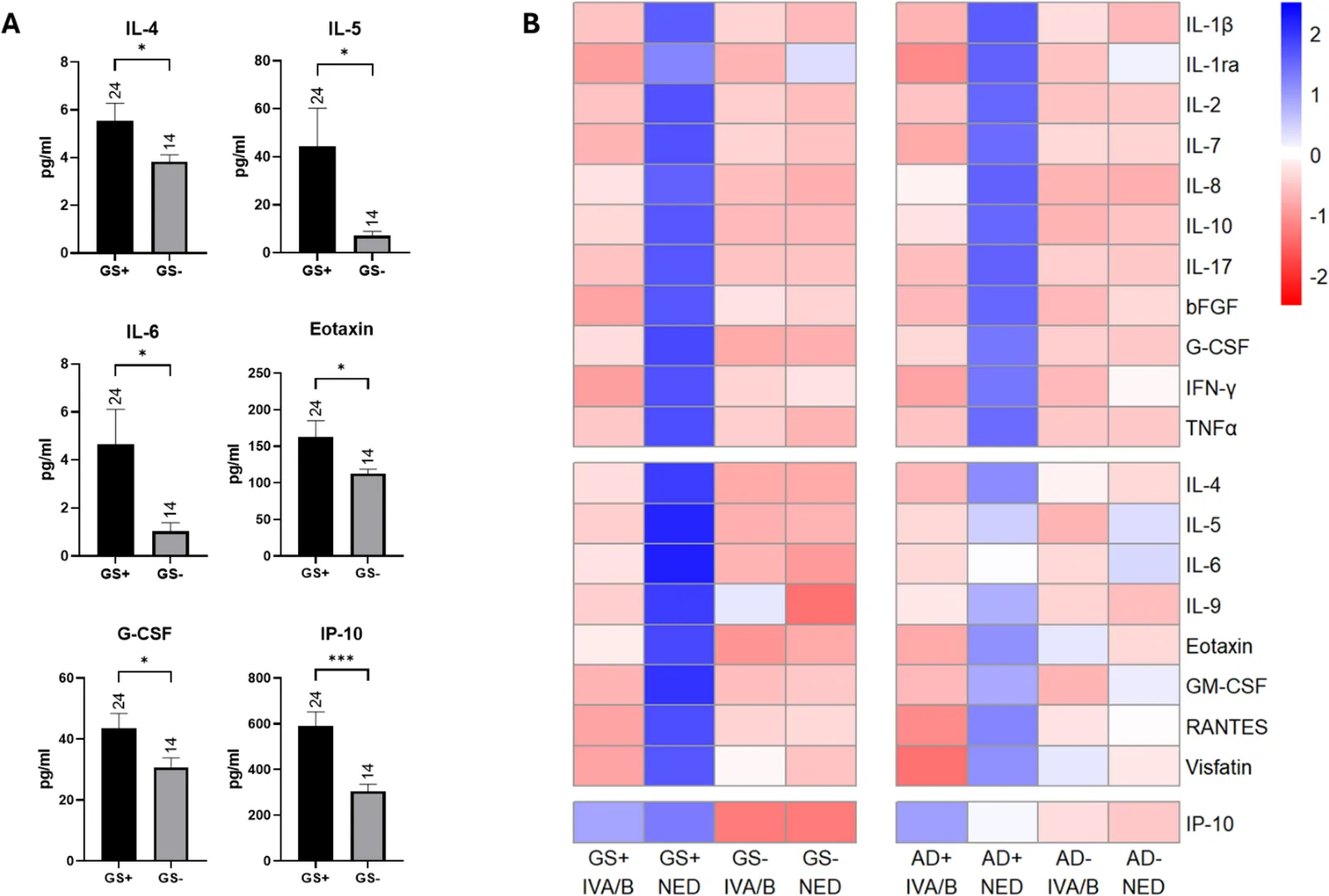
Serum cytokines in TET patients. **A** Bar plots depicting circulating levels of screened cytokines, chemokines, growth factors and metabolic markers in GS + and GS- TET patients. All concentrations are reported as mean ± SEM and expressed in pg/ml. Only molecules showing significant differences between GS + and GS- groups are shown. Asterisks denote statistical significance (Welch’s t test or Mann-Whitney U test: *p-value < 0.05; ***p-value < 0.001). **B** Heatmap depicting circulating z-scaled levels of screened cytokines, chemokines, growth factors and metabolic markers in TET patients stratified by GS (GS+/GS-), AD (AD+/AD-), and disease stage (IVA/B or NED). Mean concentrations are color-coded as follows: higher values are shown in shades of blue, lower values are shown in shades of red. Only molecules with significant comparisons between groups are shown and reported in Supplementary Tables 3 and 4.

We next evaluated circulating cytokines, chemokines, growth factors, and metabolic markers. GS+ patients displayed significantly higher levels of inflammatory mediators, including IL-4, IL-5, IL-6, Eotaxin, G-CSF, and IP-10 compared with GS- patients (Fig. 4A and Supplementary Table 2). No significant differences were observed after stratification according to AD status alone.

After stratification by disease stage, distinct patterns were found. Notably, the first cluster of inflammatory-related cytokines (IL-1β, IL-2, IL-8, IFN-γ, TNFα), immune-modulators (IL-7, IL-10, IL-17), and growth factors (bFGF, G-CSF) showed significantly higher levels in both GS + NED (Supplementary Table 3) and AD + NED (Supplementary Table 4) patients compared with all other subgroups (Fig. 4B). The second cluster of molecules (IL-4, IL-5, IL-6, IL-9, Eotaxin, GM-CSF, RANTES, and Visfatin) was predominantly increased in GS + NED patients, but not in AD + NED patients (Fig. 4B, Supplementary Tables 3–4). Among all evaluated mediators, IP-10 remained consistently elevated in GS+ patients regardless of disease stage. No differences were found for IP-10 within AD + and stage stratification (Fig. 4B, Supplementary Tables 3–4).

**Fig. 5:**
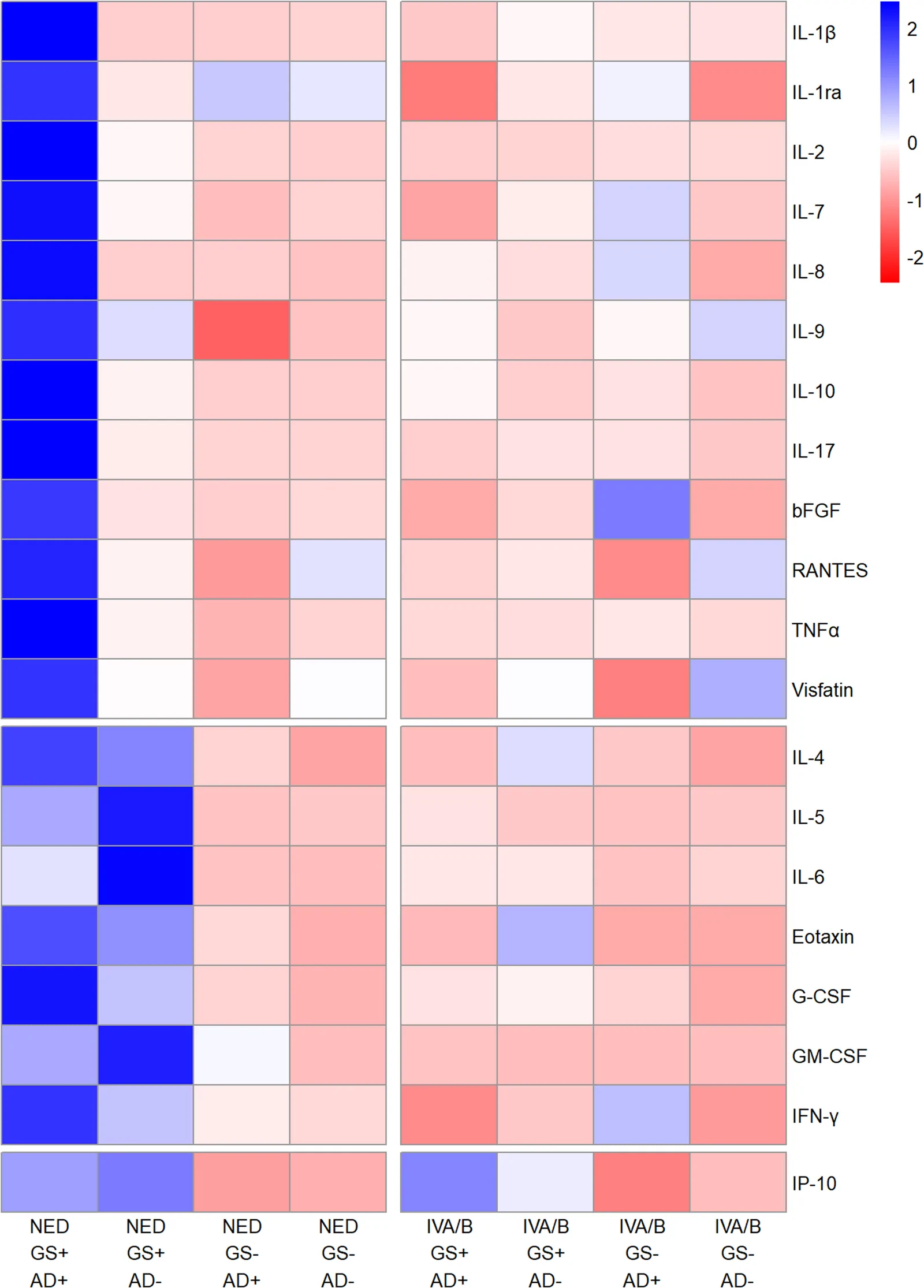
Serum cytokines are stratified by stage and AD. Heatmap depicts circulating z-scaled levels of screened cytokines, chemokines, growth factors and metabolic markers in TET patients stratified by combined GS status (GS+/GS-), AD status (AD+/AD-) and disease stage (IVA/B or NED). Values are color-coded according to the legend shown: higher values are shown in shades of blue, lower values are shown in shades of red. Only molecules showing significant differences between groups are shown

Finally, stratification according to both GS and AD status identified two major cytokine clusters. The first cluster including IL-1β, IL-1ra, IL-2, IL-7, IL-8, IL-9, IL-10, IL-17, bFGF, RANTES, TNFα, and Visfatin was predominantly enriched in GS + AD+ NED patients, compared to all other groups, both NED and IVA/B stages (Fig. 5). The second cluster including IL-4, IL-5, IL-6, Eotaxin, G-CSF, GM-CSF, and IFN-γ was significantly increased in GS + NED patients independently of AD status (Fig. 5).

## Discussion

To date the management of TETs remains challenging due to the rarity of the disease and the limited size of available patient cohorts. The high frequency of TETs associated with AD further complicates the definition of standardized clinical management and care. In this context, GS diagnosis is currently based on thymoma in association with decreased levels of IgA, IgM and IgG, peripheral B-cell lymphopenia or absence of B cells, and recurrent infections [11]. In addition, an inverted CD4+/CD8 + T cell ratio may occur together with impaired T lymphocyte responses to mitogens. Other clinical manifestations include AD onset and secondary malignancy [18]. Recurrent infections remain a major cause of mortality in patients with advanced disease, although Ig replacement therapy has significantly improved outcomes [19, 20]. We previously demonstrated more marked B-cell lymphopenia, and reduced Treg frequencies in GS + AD+ TET patients compared with GS + AD- TET patients, suggesting profound remodelling of both B- and T- cell compartments [9]. However, data regarding immune modulation across disease stages remain limited.

In this study, we provide evidence that TET patients with advanced stages (IVA/B) or NED display distinct immunological profiles according to GS and AD status, involving Ig, immune cell subsets, and circulating mediators.

GS+ patients showed reduced IgM and IgG levels compared with GS− patients; however, these differences were largely lost in advanced disease stages. Reduced IgM levels persisted only in NED patients (Fig. 1B), particularly in the absence of concomitant AD (Fig. 1C). These results suggest that serum Ig levels may be less reliable for GS identification in the metastatic stages and in the presence of concomitant AD. In contrast, peripheral B-cell lymphopenia was consistently observed in GS+ patients (Fig. 2A) across disease stages (Fig. 2C) and independently of AD status (Fig. 2E). In GS– patients, advanced disease was associated with increased B-cell lymphopenia compared with NED patients (Fig. 2C), supporting the relevance of B-cell quantification as a stable immunological marker across clinical stages.

Treg frequencies showed a stage- and context-dependent pattern. Increased Treg levels were observed in GS− patients with advanced disease compared with NED (Fig. 2D), particularly in the presence of AD (Fig. 2F), suggesting that disease progression, AD, or GS alone may independently contribute to Treg expansion. In contrast, this pattern appeared attenuated when GS and AD coexisted. Elevated Treg frequencies were also observed in GS + AD+ NED patients compared with both GS-AD + and GS + AD- NED subgroups, indicating that the coexistence of GS and AD may further influence Treg expansion levels even in the absence of disease progression. Overall, these data suggest a potential additive effect of GS and AD on Treg frequencies, with higher levels observed when both conditions coexist. Notably, Treg dynamics in GS- patients showed an opposite pattern compared with B-cell alterations (Fig. 2C, D).

In these patients, neoplastic transformation may contribute to altered processes of positive and negative thymocyte selection leading to loss of self-tolerance, as previously suggested [9]. In this context, our data further suggest that increased Treg frequencies may represent a compensatory immunoregulatory mechanism contributing to B-cell immunosuppression.

Among other immune cell subsets, monocyte levels were reduced in GS + vs. GS- patients (Fig. 3A). This effect was mainly observed in advanced IVA/B stages (Fig. 3B) and appeared influenced by the presence of AD (Fig. 3C). We previously reported similar CD4+/CD8 + ratios in GS + AD+ and GS + AD- TET patients [9]; here, we demonstrate that an inverted CD4+/CD8 + ratio was more evident in GS + AD+ patients with advanced disease, supporting a link between combined immune dysregulation and disease progression. (Fig. 3D).

Interestingly, the analysis of circulating mediators revealed up-regulation of several cytokines, chemokines and growth factors including IL-4, IL-5, IL-6, Eotaxin, G-CSF, and IP-10 in GS + TET patients (Fig. 4A). Notably, IP-10 remained consistently elevated regardless of disease stage or AD status, with particularly high levels observed in GS + NED patients (Fig. 4B, Supplementary Table 3). In stage-stratified analyses, IP-10 was the only marker persistently up-regulated in GS+ patients in both NED and IVA/B groups compared with GS- patients (Fig. 4B), suggesting a stable association with GS rather than disease stage. Previous studies have linked high levels of IP-10 in patients with AD to pro-inflammatory T-cell polarization toward Th1/Th17 responses [21]. In addition, IP-10 has been shown to amplify IL-6 production by autoreactive B cells, promoting their differentiation into IgM-, IgG-, and IgA-secreting plasma cells [22]. In this context, the cytokine profile observed in GS + NED patients (Supplementary Table 3), may reflect a sustained pro-inflammatory state associated with underlying B-cell and Ig deficiency.

Given the limited number of patients in combined GS and AD subgroups, particularly in serological analyses (Fig. 5), these observations should be considered descriptive, although supported by our previous study [9]. Indeed, we previously demonstrated increased levels of several of these mediators in GS + AD+ TET patients compared with GS + AD-TET patients [9]. In the present study, the upregulation of these and additional mediators (Supplementary Table 3, Fig. 5) observed in GS + NED patients may, in part, occur independently of AD status.

Moreover, increased IL-6 levels in GS + AD- NED patients mirrored the opposite trend observed for Treg frequencies in the same subgroup compared with GS + AD+ patients, consistent with the known immunosuppressive role of IL-6 in regulating Treg function in autoimmune conditions [23]. Conversely, higher Treg levels observed in GS + AD+ NED patients were associated with increased IL-10 levels, a cytokine involved in Treg differentiation and function [24, 25]. Interestingly, IP-10 up-regulation in GS+ patients appeared independent of concomitant AD and may, at least in NED patients, be supported by increased IFN-γ secretion (Fig. 5), according to its known regulatory axis [21, 26].

Overall, our data identify distinct but interconnected immune pathways associated with GS in TET patients, involving both regulatory and pro-inflammatory components that vary according to disease stage and AD status.

Considering the complex immune dysregulation observed in TETs and the frequent coexistence of AD, our findings identify peripheral B-cell lymphopenia and elevated circulating IP-10 levels as consistent immunological features associated with GS, including in patients with advanced-stage disease. In contrast, serum Ig levels appeared less reliable for GS identification in advanced IVA/B stages, possibly reflecting tumor-associated dysregulation. These findings support the diagnostic value of peripheral blood immunophenotyping, particularly B-cell quantification and IP-10 assessment, in clinically challenging GS/TET cases.

Future prospective studies in larger patient cohorts are warranted to validate the clinical utility of these biomarkers and to better define their role in disease monitoring and risk stratification. In addition, longitudinal immune profiling may help improve patient follow-up and support more personalized management strategies in GS-associated TET patients.

## Supplementary Information

Below is the link to the electronic supplementary material.

Supplementary Material 1

Supplementary Material 2

## Data Availability

The datasets generated and/or analyzed during the current study are available from the corresponding author on reasonable request.
