# Supplementary material for: Immunopathological Profile of Patients with Thymic Epithelial Tumour and Good Syndrome in Advanced Stage
Source: J Clin Immunol. 2026 Jul 4;46(1):91. doi: 10.1007/s10875-026-02050-w (PMC13623799; doi:10.1007/s10875-026-02050-w)
Supplement: Supplementary file 2 [file 10875_2026_2050_MOESM2_ESM.docx]

**Supplementary material**

**Figure S1. Immune cell subsets of TET patients in the presence and absence of autoimmune diseases (AD).** B cells, Treg, CD4+/CD8+ ratio are reported in histograms for AD+ and AD- TET patients and according to disease stage (IVA/B and NED). The number of patients in each group is reported above the bars. Statistical analysis was performed as detailed in Material and Methods (*p<0.05, **p<0.01).

**Supplementary Table 1: Additional treatment characteristics of TET patients.**

The table summarizes anticancer treatments for TET patients, stratified by Good syndrome status (GS+ or GS−). Anticancer treatment refers to the most recent systemic anticancer therapy administered before blood sampling. Radiotherapy data refer to treatments received during the patients’ clinical history. Data are presented as n (%). P-values refer to comparisons between GS+ and GS− patients for the overall distribution of each categorical variable and were calculated using Fisher's exact test.

| **Treatment characteristics** | **Overall (N=70)** | **GS+ (N=37)** | **GS− (N=33)** | **p-value** |
| --- | --- | --- | --- | --- |
| **Anticancer treatment prior to blood sampling, n (%)** | | | | |
| Yes | 29 (41%) | 17 (46%) | 12 (36%) | 0.472 |
| No | 41 (59%) | 20 (54%) | 21 (64%) |  |
| **Anticancer treatment type, n (%)** | | | | |
| Etoposide | 9 (13%) | 6 (16%) | 3 (9%) | 0.280 |
| SSA | 11 (16%) | 6 (16%) | 5 (15%) |  |
| SSA + Etoposide | 3 (4%) | 0 (0%) | 3 (9%) |  |
| Platinum-based | 2 (3%) | 1 (3%) | 1 (3%) |  |
| Gemcitabine + Capecitabine | 1 (1%) | 1 (3%) | 0 (0%) |  |
| Capecitabine + Etoposide | 1 (1%) | 1 (3%) | 0 (0%) |  |
| Pembrolizumab + Etoposide | 2 (3%) | 2 (5%) | 0 (0%) |  |
| **Radiotherapy (clinical history), n (%)** | | | | |
| Adjuvant | 15 (21%) | 10 (27%) | 5 (15%) | 0.478 |
| Palliative | 5 (7%) | 2 (5%) | 3 (9%) |  |
| None | 50 (71%) | 25 (68%) | 25 (76%) |  |

| **Supplementary Table 2: Serum markers according to Good Syndrome and autoimmune disease status.**  The table shows circulating levels of cytokines, chemokines, growth factors and metabolic markers in TET patients stratified according to Good syndrome (GS+/GS-) and autoimmune disease status (AD+/AD-). All variables are reported as mean (SEM) and expressed as pg/ml. P-values reported in bold are considered statistically significant (p-value <0.05). | | | | | | |
| --- | --- | --- | --- | --- | --- | --- |
|  | **Good Syndrome** | | | **Autoimmune Diseases** | | |
|  | **GS+ (n: 24)** | **GS- (n: 14)** | **p-value** | **AD+ (n: 19)** | **AD- (n: 19)** | **p-value** |
| **IL-1β** | 1.81 (0.57) | 1.23 (0.16) | 0.447 | 1.83 (0.72) | 1.36 (0.74) | 0.532 |
| **IL-1ra** | 334 (55.1) | 380 (70.3) | 0.613 | 340 (61.6) | 363 (61.4) | 0.796 |
| **IL-2** | 3.75 (2.15) | 1.52 (0.2) | 0.310 | 4.21 (2.71) | 1.64 (0.29) | 0.351 |
| **IL-4** | 5.54 (0.74) | 3.83 (0.29) | **0.04** | 4.92 (0.73) | 4.91 (0.68) | 0.993 |
| **IL-5** | 44.3 (15.9) | 7.16 (1.84) | **0.029** | 33.4 (11) | 27.9 (17.9) | 0.797 |
| **IL-6** | 4.66 (1.45) | 1.02 (0.36) | **0.022** | 2.99 (0.77) | 3.65 (1.79) | 0.739 |
| **IL-7** | 10.8 (2.21) | 9.72 (1.03) | 0.66 | 10.8 (2.72) | 10 (1.02) | 0.786 |
| **IL-8** | 26.9 (5.14) | 18.5 (3.53) | 0.185 | 29.4 (6.37) | 18.1 (2.65) | 0.115 |
| **IL-9** | 529 (13.8) | 514 (13.5) | 0.434 | 530 (16.5) | 517 (11.5) | 0.535 |
| **IL-10** | 3.69 (1.43) | 1.22 (0.27) | 0.102 | 4.27 (1.79) | 1.29 (0.22) | 0.115 |
| **IL-17** | 8.09 (3.52) | 3.98 (0.39) | 0.258 | 8.46 (4.43) | 4.69 (0.7) | 0.412 |
| **Eotaxin** | 163 (21.9) | 112 (6.22) | **0.035** | 138 (17.9) | 151 (23.2) | 0.642 |
| **bFGF** | 24.4 (3.3) | 24.7 (2.67) | 0.932 | 26.2 (4.48) | 22.9 (0.99) | 0.482 |
| **G-CSF** | 43.6 (4.79) | 30.8 (3.06) | **0.031** | 42.5 (5.88) | 35.2 (3.2) | 0.282 |
| **GM-CSF** | 3.21 (1.36) | 1.62 (0.61) | 0.294 | 2.74 (1.1) | 2.51 (1.43) | 0.898 |
| **IFNγ** | 8.16 (1.48) | 8.36 (1.28) | 0.917 | 8.48 (1.76) | 7.98 (1.13) | 0.814 |
| **IP-10** | 591 (61.9) | 306 (30.2) | **<0.001** | 558 (81.3) | 414 (39.7) | 0.125 |
| **MCP-1** | 66.6 (12) | 64.9 (11.5) | 0.922 | 67.8 (14.5) | 64.1 (9.52) | 0.831 |
| **MIP-1α** | 5.02 (1.24) | 2.9 (0.41) | 0.117 | 5.47 (1.55) | 3.01 (0.31) | 0.137 |
| **MIP-1β** | 306 (9.35) | 302 (8.11) | 0.749 | 312 (10.7) | 297 (7.44) | 0.269 |
| **PDGF** | 460 (107) | 575 (140) | 0.519 | 452 (127) | 553 (114) | 0.557 |
| **RANTES** | 3924 (259) | 3891 (279) | 0.932 | 3827 (328) | 3997 (203) | 0.663 |
| **TNFα** | 44.8 (4.9) | 37.2 (1.72) | 0.152 | 45.4 (6.24) | 38.6 (1.26) | 0.296 |
| **C-Peptide** | 1227 (150) | 1438 (207) | 0.415 | 1156 (127) | 1454 (204) | 0.225 |
| **Ghrelin** | 351 (49.6) | 273 (42) | 0.237 | 282 (52.8) | 363 (45.9) | 0.253 |
| **GIP** | 142 (27.1) | 154 (33) | 0.782 | 128 (32.1) | 165 (26.4) | 0.378 |
| **GLP-1** | 373 (22.7) | 349 (21) | 0.43 | 363 (26.2) | 366 (19.8) | 0.93 |
| **Glucagon** | 2485 (115) | 2709 (160) | 0.266 | 2492 (129) | 2643 (137) | 0.43 |
| **Insulin** | 439 (82.3) | 599 (168) | 0.401 | 459 (87.8) | 537 (137) | 0.637 |
| **Leptin** | 5704 (1118) | 6817 (1876) | 0.615 | 5794 (1165) | 6433 (1598) | 0.749 |
| **PAI-1** | 4953 (213) | 4758 (404) | 0.672 | 4829 (236) | 4934 (324) | 0.795 |
| **Resistin** | 9710 (920) | 10095 (1353) | 0.816 | 8907 (960) | 10796 (1150) | 0.216 |
| **Visfatin** | 1002 (293) | 990 (269) | 0.976 | 803 (308) | 1192 (279) | 0.355 |

| **Supplementary Table 3: Serum markers according to Good syndrome status and disease stage.**  The table shows circulating levels of cytokines, chemokines, growth factors and metabolic markers in TET patients stratified according to the presence or absence of Good syndrome (GS+/GS-) and disease stage (IVA/B or NED). All variables are reported as mean (SEM) and expressed as pg/ml. Overall p-values reported in bold are considered statistically significant (p-value <0.05). **^a^** denotes significant difference versus the GS+NED group; **^b^** denotes significant difference versus the GS+IVA/B group. | | | | | |
| --- | --- | --- | --- | --- | --- |
|  | **GS+** | | **GS-** | | **Overall p-value** |
|  | **IVA/B (n: 19)** | **NED**  **(n: 5)** | **IVA/B**  **(n: 6)** | **NED**  **(n: 8)** |  |
| **IL-1β** | 1.22 (0.16) **^a^** | 4.05 (2.63) | 1.43 (0.32) **^a^** | 1.08 (0.16) **^a^** | **0.034** |
| **IL-1ra** | 281 (44.3) **^a^** | 538 (193) | 306 (56.9) **^a^** | 436 (115) | **0.037** |
| **IL-2** | 1.33 (0.15) **^a^** | 13 (10) | 1.84 (0.34) **^a^** | 1.27 (0.22) **^a^** | **0.033** |
| **IL-4** | 4.77 (0.73) **^a^** | 8.49 (1.81) | 3.83 (0.34) **^a^** | 3.83 (0.47) **^a^** | **0.026** |
| **IL-5** | 19.7 (5.79) **^a^** | 138 (60) | 6.83 (1.47) **^a^** | 7.4 (3.13) **^a^** | **<0.001** |
| **IL-6** | 3.02 (0.61) **^a^** | 10.9 (6.3) | 1.47 (0.75) **^a^** | 0.68 (0.29) **^a^** | **0.01** |
| **IL-7** | 8.2 (1.24) **^a^** | 20.7 (8.8) | 9.72 (1.03) **^a^** | 8.99 (0.86) **^a^** | **0.015** |
| **IL-8** | 22.8 (4.8) | 42.4 (16.1) | 19.2 (4.48) **^a^** | 17.9 (5.45) **^a^** | 0.057 |
| **IL-9** | 518 (14.6) **^a^** | 571 (32.4) | 534 (20.2) | 498 (17) **^a^** | 0.061 |
| **IL-10** | 2.23 (0.68) **^a^** | 9.24 (6.2) | 1.17 (0.49) **^a^** | 1.26 (0.34) **^a^** | **0.046** |
| **IL-17** | 4.26 (0.77) **^a^** | 22.7 (16.3) | 3.91 (0.67) **^a^** | 4.03 (0.5) **^a^** | **0.042** |
| **Eotaxin** | 145 (24.2) **^a^** | 233 (41.4) | 108 (5.35) **^a^** | 116 (10.4) **^a^** | **0.047** |
| **bFGF** | 20.7 (1.48) **^a^** | 38.3 (14.2) | 24.7 (2.67) **^a^** | 23.6 (1.12) **^a^** | **<0.001** |
| **G-CSF** | 37.7 (4.16) **^a^** | 66 (13.4) | 30.8 (3.06) **^a^** | 30.9 (3.51) **^a^** | **0.002** |
| **GM-CSF** | 1.25 (0.42) **^a^** | 10.7 (5.54) | 1.62 (0.61) **^a^** | 1.94 (1.04) **^a^** | **<0.001** |
| **IFNγ** | 5.81 (0.81) **^a^** | 17.1 (4.86) | 8.04 (1.9) **^a^** | 8.61 (4.25) **^a^** | **0.003** |
| **IP-10** | 581 (77.1) | 630 (468) | 305 (155) **^a,b^** | 308 (228) **^a,b^** | **0.023** |
| **MCP-1** | 64.5 (15) | 74.6 (8.97) | 67.3 (26) | 63.1 (22.9) | 0.983 |
| **MIP-1α** | 5.18 (1.56) | 4.4 (0.91) | 2.49 (0.5) | 3.22 (0.62) | 0.641 |
| **MIP-1β** | 301 (10.5) | 325 (20.2) | 316 (8.14) | 291 (11.8) | 0.426 |
| **PDGF** | 355 (103) | 859 (291) | 548 (134) | 595 (232) | 0.248 |
| **RANTES** | 3665 (152) **^a^** | 4911 (1069) | 3876 (462) **^a^** | 3903 (373) | **0.002** |
| **TNFα** | 38.2 (1.82) **^a^** | 70.1 (20.1) | 39 (1.98) **^a^** | 35.9 (2.62) **^a^** | **0.004** |
| **C-Peptide** | 1329 (180) | 839 (129) | 1708 (428) | 1236 (842) | 0.293 |
| **Ghrelin** | 355 (55.1) | 335 (126) | 179 (50.7) | 343 (51.9) | 0.378 |
| **GIP** | 138 (26.9) | 154 (88.2) | 139 (46.8) | 164 (48.2) | 0.969 |
| **GLP-1** | 370 (20.6) | 385 (82.7) | 401 (32.2) | 309 (19.1) | 0.329 |
| **Glucagon** | 2465 (137) | 2563 (200) | 2423 (164) | 2923 (231) | 0.266 |
| **Insulin** | 496 (99.9) | 220 (34.7) | 806 (378) | 444 (86.5) | 0.269 |
| **Leptin** | 5560 (1318) | 6248 (2166) | 3613 (1247) | 9220 (2946) | 0.355 |
| **PAI-1** | 4845 (263) | 5364 (154) | 5146 (242) | 4466 (686) | 0.589 |
| **Resistin** | 9687 (1008) | 9797 (2443) | 10275 (3057) | 9961 (967) | 0.995 |
| **Visfatin** | 742 (253) **^a^** | 1988 (980) | 1138 (579) | 879 (228) **^a^** | **0.044** |

| **Supplementary Table 4: Serum markers in Autoimmune Diseases and disease stage**.  The table shows circulating levels of cytokines, chemokines, growth factors and metabolic markers in patients with or without Autoimmune Diseases (AD+/AD-) and stratified as stage IVA or B (IVA/B) or with no evidence of disease (NED). All variables are reported as mean (SEM) and expressed as pg/ml. Overall p-values reported in bold are considered statistically significant (p-value <0.05). **^a^** denotes significant difference versus the AD+NED group. | | | | | |
| --- | --- | --- | --- | --- | --- |
|  | **AD+** | | **AD-** | | **Overall p-value** |
|  | **IVA/B (n: 14)** | **NED**  **(n: 5)** | **IVA/B**  **(n: 11)** | **NED**  **(n: 8)** |  |
| **IL-1β** | 1.04 (0.13) **^a^** | 4.04 (2.64) | 1.57 (0.26) **^a^** | 1.09 (0.14) **^a^** | **<0.001** |
| **IL-1ra** | 256 (26.9) **^a^** | 576 (198) | 327 (74.3) **^a^** | 412 (108) | **0.02** |
| **IL-2** | 1.39 (0.19) **^a^** | 12.1 (10.2) | 1.53 (0.23) **^a^** | 1.79 (0.64) **^a^** | **0.013** |
| **IL-4** | 4.1 (0.57) | 7.2 (2.11) | 5.11 (1.05) | 4.64 (0.79) | 0.274 |
| **IL-5** | 23.3 (7.57) | 61.6 (35.7) | 8.16 (1.99) | 55 (42.1) | 0.282 |
| **IL-6** | 2.67 (0.71) | 3.89 (2.32) | 2.63 (0.75) | 5.05 (4.24) | 0.804 |
| **IL-7** | 7.76 (1.38) **^a^** | 19.3 (9.18) | 10.1 (1.69) **^a^** | 9.84 (0.91) | **0.012** |
| **IL-8** | 24.7 (6.35) **^a^** | 42.6 (16.4) | 18.4 (2.99) **^a^** | 17.8 (5.03) **^a^** | **0.041** |
| **IL-9** | 524 (18.2) | 546 (39.4) | 519 (15.2) | 514 (18.5) | 0.834 |
| **IL-10** | 2.71 (0.91) **^a^** | 8.66 (6.4) | 1.04 (0.21) **^a^** | 1.62 (0.44) **^a^** | **0.028** |
| **IL-17** | 3.73 (0.59) **^a^** | 21.7 (16.5) | 4.73 (1.16) **^a^** | 4.64 (0.61) **^a^** | **0.021** |
| **Eotaxin** | 115 (14.8) | 201 (46.1) | 163 (37.3) | 136 (22) | 0.259 |
| **bFGF** | 22.1 (3.2) **^a^** | 37.5 (14.4) | 22 (1.43) **^a^** | 24.1 (1.29) **^a^** | **0.038** |
| **G-CSF** | 36.4 (5.05) **^a^** | 59.7 (16.1) | 35.5 (4.78) **^a^** | 34.8 (4.17) **^a^** | **0.035** |
| **GM-CSF** | 1.33 (0.52) | 6.69 (3.6) | 1.12 (0.4) | 4.42 (3.36) | 0.156 |
| **IFNγ** | 5.93 (1.02) **^a^** | 15.6 (5.12) | 6.87 (1.22) **^a^** | 9.52 (2.09) **^a^** | **0.018** |
| **IP-10** | 586 (105) | 479 (98.1) | 423 (52) | 402 (65.6) | 0.411 |
| **MCP-1** | 69.1 (19.2) | 64.4 (14.7) | 60.2 (12.3) | 69.5 (15.7) | 0.976 |
| **MIP-1α** | 5.81 (2.1) | 4.5 (0.92) | 2.9 (0.35) | 3.15 (0.59) | 0.476 |
| **MIP-1β** | 312 (12.9) | 312 (21.6) | 296 (9.15) | 299 (13.1) | 0.748 |
| **PDGF** | 363 (133) | 701 (307) | 450 (100) | 694 (233) | 0.417 |
| **RANTES** | 3536 (181) | 4644 (1145) | 3944 (262) | 4070 (341) | 0.326 |
| **TNFα** | 38.1 (2.33) **^a^** | 65.8 (21.8) | 38.7 (1.56) **^a^** | 38.5 (2.21) **^a^** | **0.03** |
| **C-Peptide** | 1168 (165) | 1122 (164) | 1741 (306) | 1060 (181) | 0.144 |
| **Ghrelin** | 293 (67.6) | 250 (75.2) | 338 (61.2) | 397 (72.1) | 0.626 |
| **GIP** | 117 (30.2) | 156 (94.9) | 166 (34.7) | 163 (43.4) | 0.780 |
| **GLP-1** | 367 (22) | 351 (85.2) | 391 (28.5) | 330 (22.5) | 0.629 |
| **Glucagon** | 2376 (124) | 2818 (327) | 2555 (195) | 2764 (192) | 0.343 |
| **Insulin** | 493 (116) | 364 (80.9) | 669 (223) | 354 (91.3) | 0.519 |
| **Leptin** | 4524 (1059) | 9351 (2950) | 5818 (2008) | 7280 (2738) | 0.91 |
| **PAI-1** | 4694 (308) | 5207 (209) | 5202 (251) | 4565 (696) | 0.593 |
| **Resistin** | 8751 (975) | 9346 (2655) | 11199 (1953) | 10242 (712) | 0.628 |
| **Visfatin** | 480 (153) | 1707 (1063) | 1292 (471) | 1055 (189) | 0.221 |
